# Tocilizumab for Advanced Non‐Small‐Cell Lung Cancer With Concomitant Cachexia: An Observational Study

**DOI:** 10.1002/jcsm.13638

**Published:** 2024-11-11

**Authors:** Yang Du, Xiao‐Yan Liu, Rui‐Li Pan, Xiao‐Tong Zhang, Xiao‐Yan Si, Min‐Jiang Chen, Meng‐Zhao Wang, Li Zhang

**Affiliations:** ^1^ Department of Respiratory and Critical Care Medicine, Peking Union Medical College Hospital Chinese Academy of Medical Sciences & Peking Union Medical College Beijing China

**Keywords:** cachexia, interleukin‐6, non‐small‐cell lung cancer, tocilizumab

## Abstract

**Background:**

Cancer cachexia significantly contributes to morbidity and mortality in patients with non‐small‐cell lung cancer (NSCLC). Inflammatory pathways mediated by interleukin‐6 (IL‐6) play a crucial role in the development of cancer cachexia. This study aimed to investigate the use of tocilizumab in the management of NSCLC with coexisting IL‐6‐elevated cachexia.

**Methods:**

In this retrospective study, data were collected from patients with NSCLC and concurrent IL‐6‐elevated cachexia who received either tocilizumab plus antitumour therapy or antitumour therapy alone. The primary endpoints were overall survival (OS) and improved modified Glasgow Prognostic Score (mGPS) at Week 12. The secondary endpoints included changes from baseline over 12 weeks in body weight, albumin, C‐reactive protein (CRP) and mGPS. Qualitative improvements in patient self‐rated appetite and fatigue were reported as exploratory analysis.

**Results:**

The study included 49 patients diagnosed with NSCLC and IL‐6‐elevated cachexia, Eastern Cooperative Oncology Group performance status of 2–4. Of these, 26 received tocilizumab in combination with antitumour therapy, and 23 received antitumour therapy alone. The majority of these patients were male (87.8%). Baseline characteristics were almost identical between the two groups. The tocilizumab group demonstrated a significantly longer median OS compared to the control group (15.1 vs. 3.2 months; hazard ratio 0.18, 95% confidence interval 0.08–0.38; *p* < 0.001). The rate of patients surviving with mGPS improvement at Week 12 was significantly higher in the tocilizumab group than in the control group (risk difference 0.88, 95% confidence interval 0.75–1.00; *p* < 0.001). Over the 12‐week period, significant improvements were observed in body weight, albumin, CRP and mGPS in the tocilizumab group compared to the control group (body weight: 5.15 ± 0.53 kg vs. −5.69 ± 0.76 kg, *p* = 0.041; albumin: 5.89 ± 0.70 g/L vs. −2.97 ± 0.71 g/L, *p* < 0.001; CRP: −91.50 ± 7.15 mg/L vs. 9.47 ± 13.69 mg/L, *p* < 0.001; mGPS: −1.61 ± 0.15 vs. 0.03 ± 0.08, *p* < 0.001). The tocilizumab group also displayed significantly higher rates of improvement in appetite and fatigue (both *p* < 0.001). The incidence of Grade 3 or higher adverse events was 34.6% in the tocilizumab group compared to 78.3% in the control group. Tocilizumab‐related adverse events were observed in three patients (11.5%), including two cases of neutropenia and one case of skin and subcutaneous tissue infection.

**Conclusion:**

Tocilizumab demonstrated significant benefits in survival and various clinical parameters, including body weight, albumin, CRP, mGPS and symptom burden in patients with NSCLC and concurrent IL‐6‐elevated cachexia. Given the existing unmet medical need for effective interventions for cancer cachexia, tocilizumab may be considered as a potential treatment option.

## 1 Background

Cancer cachexia, a multifactorial syndrome characterized by severe weight loss and muscle wasting, afflicts a large proportion of patients with non‐small‐cell lung cancer (NSCLC) and profoundly diminish their therapeutic options and survival prospects [1, 2]. Despite numerous studies focusing on nutritional support and symptomatic pharmacotherapy, there remains a conspicuous absence of drugs specifically designed to improve the prognosis of cancer cachexia [3, 4]. The development of effective pharmacological treatments for this condition is a recognized priority in oncological care.

The role of systemic inflammation, especially mediated by cytokines like interleukin‐6 (IL‐6), in the pathogenesis of cachexia in NSCLC patients, has been increasingly recognized [5, 6]. Besides, elevated plasma IL‐6 levels have been consistently associated with the resistance to antitumour treatment and poorer prognoses in NSCLC, suggesting its potential as a therapeutic target, especially for NSCLC patients with cachexia and elevated plasma IL‐6 levels [7, 8].

Tocilizumab, an antibody targeting the IL‐6 receptor, has been shown to mitigate systemic inflammation and arrest or reverse the catabolic processes central to cachexia [9, 10, 11, 12]. Also, tocilizumab could reverse chemotherapeutic resistance, augment antitumour immune responses and limit autoimmune adverse effects of immunotherapy [13, 14, 15, 16]. Consequently, tocilizumab appears to be a promising therapeutic agent for patients afflicted with cachexia. To further explore this potential, a retrospective analysis has been conducted to evaluate the efficacy and safety profile of tocilizumab in improving the prognosis of patients with cachexia and elevated plasma IL‐6 levels. This analysis is crucial in addressing the urgent need for effective treatment modalities in this patient population.

## 2 Methods

### 2.1 Study Design and Patients

This investigation was a single‐centre, retrospective, observational cohort study, focused on assessing the efficacy and safety of tocilizumab in individuals with NSCLC complicated by cachexia and elevated plasma IL‐6 levels. The cohort comprised individuals treated at the Lung Cancer Centre of Peking Union Medical College Hospital from 1 March 2019 to 30 June 2023. Eligibility criteria included adults aged 18 years or older, a histopathological diagnosis of unresectable Stage III or IV NSCLC and specific clinical manifestations. These manifestations encompassed involuntary weight loss of ≥ 5% within the preceding 6 months, cancer‐related anorexia and fatigue rated Grade 1 or higher as per the National Cancer Institute's Common Terminology Criteria for Adverse Events (CTCAE) version 5.0, systemic inflammation indicated by C‐reactive protein levels ≥ 50 mg/L and IL‐6 concentrations exceeding twice the upper limit of normal (ULN) and an Eastern Cooperative Oncology Group performance status (ECOG PS) ranging from 2 to 4. Exclusion criteria involved weight loss attributable to noncancerous causes such as gastrointestinal obstructions, infections, chronic kidney disease or autoimmune disorders; current use of appetite stimulants or corticosteroids; inflammation primarily due to factors other than cancer, such as infection or pre‐existing autoimmune conditions; the occurrence of immune‐related adverse events; and significant deficiencies in medical records. More details on the diagnosis of IL‐6‐elevated cachexia in clinical practice were described in Figure S1.

### 2.2 Ethical Considerations and Patient Consent

The study was conducted in strict accordance with the ethical principles of the Declaration of Helsinki. Tocilizumab was provided through an off‐label compassionate medication usage programme at Peking Union Medical College Hospital. The off‐label use of tocilizumab was approved by the joint session of the medical committee, pharmacy management and therapeutics committee and institutional review board of Peking Union Medical College Hospital. Informed consent was obtained from all the patients receiving tocilizumab therapy. Patient data and treatment information were collected with the approval of the institutional review board (No. I‐23PJ769).

### 2.3 Exposures

Study exposures included tocilizumab in combination with standard antitumour therapy (tocilizumab group) or standard antitumour therapy alone (control group). Tocilizumab was administered intravenously at a dose of 6–8 mg/kg over 1 h every 3 weeks, initiated within 1‐week postdiagnosis of IL‐6‐elevated cachexia. Dose adjustments or interruptions were made based on treatment‐related adverse events or at the treating oncologist's discretion. No participants in the control group received tocilizumab or other therapies targeting IL‐6 signalling. All participants received standard antitumour therapy, tailored to tumour histology and stage. Nutritional assessment, dietary counselling and oral nutritional supplements were offered to all patients by the nutrition support team, but tube feeding and parenteral nutrition were not provided unless necessary.

### 2.4 Endpoints and Evaluations

The study's primary endpoints were overall survival (OS) and improvement in the modified Glasgow Prognostic Score (mGPS) at 12 weeks. OS was calculated from the time of inflammatory cachexia diagnosis to death from any cause or the last follow‐up. The mGPS was defined as: a score of 2 for elevated C‐reactive protein (> 10 mg/L) and hypoalbuminemia (< 35 g/L), a score of 1 for elevated C‐reactive protein only and a score of 0 for absence of these abnormalities [17]. Secondary endpoints included changes in body weight, serum albumin, C‐reactive protein levels and mGPS over a 12‐week period from baseline. Qualitative improvements in self‐reported appetite and fatigue using the MD Anderson Symptom Inventory numeric rating scale were also explored. Adverse events were classified according to the Medical Dictionary for Regulatory Activities (MedDRA; version 20.0) and graded using the CTCAE (version 5.0). Follow‐up assessments were conducted at 3‐week intervals, concluding on 30 September 2023.

### 2.5 Statistical Analyses

Demographic and baseline characteristics of the participants were systematically summarized using descriptive statistics. The comparison between the tocilizumab and control groups was conducted using appropriate statistical tests, including the Wilcoxon rank‐sum test for continuous variables and Pearson's *χ* ^2^ test or Fisher's exact test for categorical variables. OS was estimated utilizing the Kaplan–Meier method, and differences between groups were assessed using the log‐rank test. Hazard ratios (HRs) for death, along with 95% confidence intervals (CIs), were derived using Cox proportional hazards models. For evaluating the survival with mGPS improvement at Week 12, risk difference (RD) and 95% CIs were calculated using bootstrap method.

Subgroup analyses, stratified by factors such as weight loss, baseline mGPS, ECOG PS, histological type, the number of prior antitumour therapy regimens and concurrent PD‐(L)1 inhibitor therapy, were performed using stratified Cox and logistic regression models for the primary endpoints. Additionally, stepwise Cox and logistic regression analyses were also employed to identify significant prognostic factors among the variables, including tocilizumab therapy, weight loss, baseline mGPS, ECOG PS, histological type, number of prior antitumour therapy regimens and concurrent PD‐(L)1 inhibitor therapy.

For secondary endpoints, a mixed‐effects model for repeated measures, considering treatment group and time point as fixed factors and baseline as a covariate, was used to analyse efficacy parameters. Least‐squares means, standard errors and 95% CIs were calculated for comparisons between groups. The analysis did not impute missing values for these secondary endpoints. Instead, a pattern mixture repeated measures model was used to address missing data, minimizing bias by treating death as missing data and analysing data from both missing and living patients separately. Safety parameters were assessed using descriptive statistics, presented as counts and percentages of patients experiencing adverse events. Statistical significance was set at a *p* value of less than 0.05. All analyses were conducted using R software version 4.2.1 (R Project for Statistical Computing) and Stata 17 (StataCorp LP, College Station, TX, USA).

## 3 Results

### 3.1 Patients and Treatment

**FIGURE 1:**
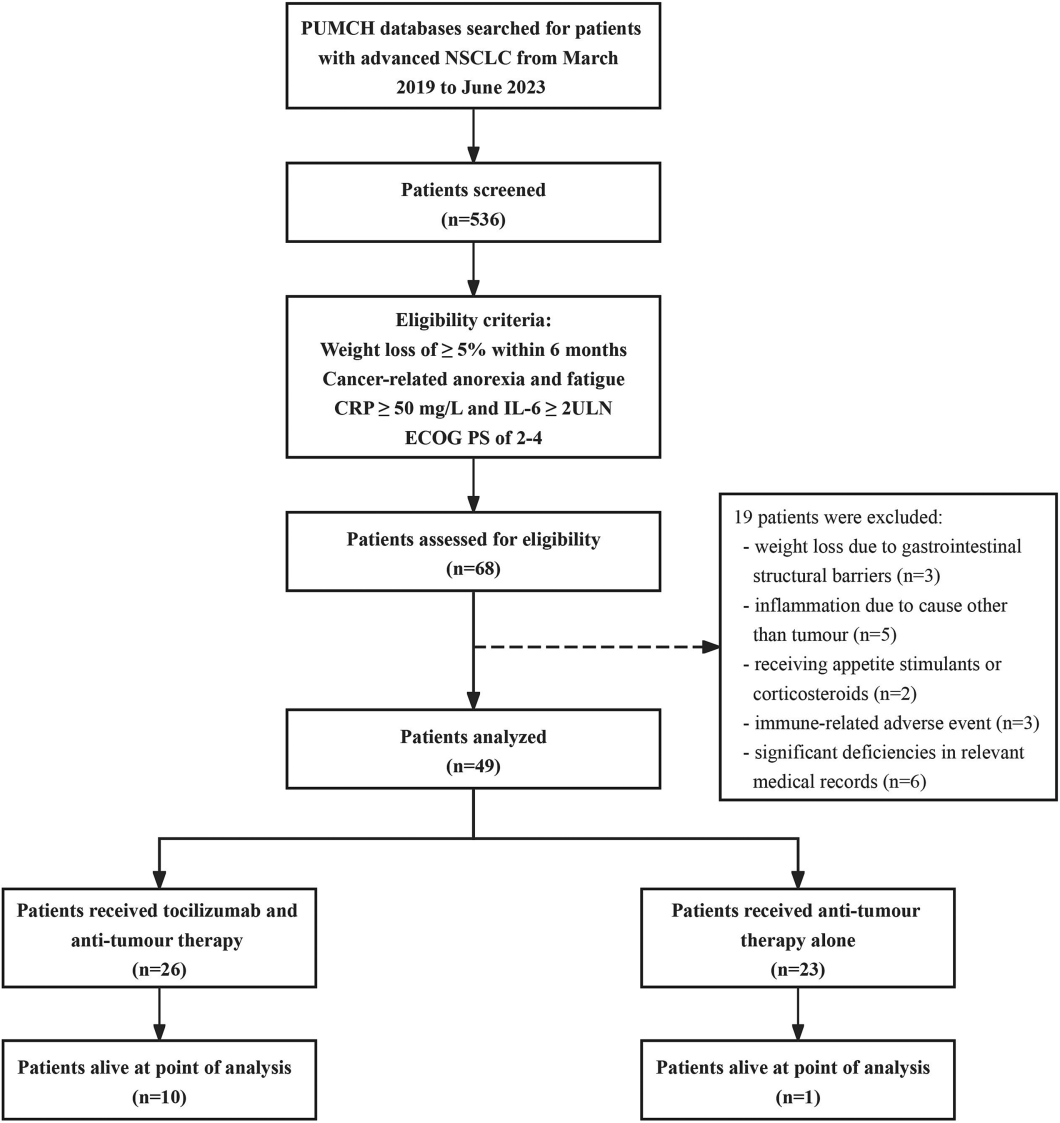
Flow diagram.

Our study included a total of 49 patients with NSCLC and IL‐6‐elevated cachexia, treated at our institution from March 2019 to September 2023 (Figure 1). The cohort comprised 26 patients (53.1%) in the tocilizumab plus antitumour therapy group and 23 patients (46.9%) in the control group. All patients presented with an ECOG PS ranging from 2 to 4. Thirty‐one (63.3%) patients experienced a weight loss exceeding 10% and 40 (81.6%) exhibited an mGPS of 2. Baseline characteristics, with the exception of body weight and BMI, were comparable between groups (Table 1). The median follow‐up duration was 15.2 months (interquartile range [IQR]: 9.5–32.7 months). In the tocilizumab group, the median number of tocilizumab doses was four (range: 1–9 doses), with a median cumulative dose of 21.0 mg/kg. As of the data cut‐off, three patients in the tocilizumab group continued tocilizumab therapy. The primary reason for discontinuation of tocilizumab was assessed amelioration of inflammatory cachexia, as evaluated by oncologists. All patients received concurrent antitumour therapies during the treatment period: most patients received chemotherapy and more than half of all patients received PD‐(L)1 inhibitor therapy. The details of concurrent antitumour regimens were described in Tables 1 and S1.

**TABLE 1:** Demographics and baseline characteristics of the patients.

	Combination of tocilizumab and antitumour therapy (*n* = 26)	Antitumour therapy alone (*n* = 23)	*p*
Age (years)			0.41
Median (range)	66 (61, 72)	69 (62, 74)	
Sex			0.56
Male	24 (92.3%)	19 (82.6%)	
Female	2 (7.7%)	4 (17.4%)	
Weight (kg)	52.5 (51.0, 61.7)	65.0 (55.0, 70.0)	0.002
BMI (kg/m^2^)	18.4 (17.4, 20.7)	22.0 (20.0, 23.3)	0.002
Weight loss			0.48
5%–10%	11 (42.3%)	7 (30.4%)	
> 10%	15 (57.7%)	16 (69.6%)	
Interleukin‐6 (pg/mL)	56.7 (43.0, 71.2)	47.6 (28.7, 143.8)	0.41
Albumin (g/L)	31 (29, 34)	30 (29, 32)	0.51
CRP (mg/L)	102.0 (85.0, 120.0)	118.1 (100.2, 164.2)	0.07
mGPS			0.58
1	6 (23.1%)	3 (13.0%)	
2	20 (76.9%)	20 (87.0%)	
ECOG PS			0.57
2	15 (57.7%)	16 (69.6%)	
3	10 (38.5%)	5 (21.7%)	
4	1 (3.8%)	2 (8.7%)	
NSCLC type per histological criteria			0.47
Adenocarcinoma	13 (50.0%)	7 (30.4%)	
Squamous cell	9 (34.6%)	12 (52.2%)	
Other	4 (15.4%)	4 (17.4%)	
Disease stage			0.95
III	2 (7.7%)	2 (8.7%)	
IV	24 (92.3%)	21 (91.3%)	
Driver mutation type			
EGFR mutation	3 (11.5%)	4 (17.4%)	0.70
KRAS mutation	1 (3.8%)	2 (8.7%)	0.59
BRAF mutation	0 (0)	1 (4.3)	0.47
PD‐L1 expression level			0.83
< 1%	24 (92.3%)	19 (82.6%)	
1%–49%	0 (0)	2 (8.7%)	
≥ 50%	2 (7.7%)	2 (8.7%)	
Number of prior antitumour therapy regimens			0.09
0	15 (57.7%)	8 (34.8%)	
1	8 (30.8%)	8 (34.8%)	
> 1	3 (11.5%)	7 (30.4%)	
Concurrent antitumour regimens			
Chemotherapy monotherapy	0 (0)	5 (21.7%)	0.02
PD‐(L)1 inhibitor monotherapy	1 (3.8%)	1 (4.3%)	1.0
Target monotherapy	2 (7.7%)	2 (8.7%)	1.0
Chemoimmunotherapy	16 (61.5%)	10 (43.5%)	0.26
Chemotherapy plus targeted therapy	4 (15.4%)	3 (13.0%)	1.0
Immunotarget therapy	1 (3.8%)	0 (0)	1.0
Chemoimmunotherapy plus targeted therapy	2 (7.7%)	1 (4.3%)	1.0

Abbreviations: BMI, body mass index; BRAF, vrafmurine sarcoma viral oncogene homologue B; CRP, C‐reactive protein; ECOG PS, Eastern Cooperative Oncology Group performance status; EGFR, epidermal growth factor receptor; KRAS, Kirsten rats arcomaviral oncogene homologue; mGPS, modified Glasgow Prognostic Score; NSCLC, non–small‐cell lung cancer; PD‐(L)1, programmed cell death protein (ligand) 1.

### 3.2 Efficacy

**FIGURE 2:**
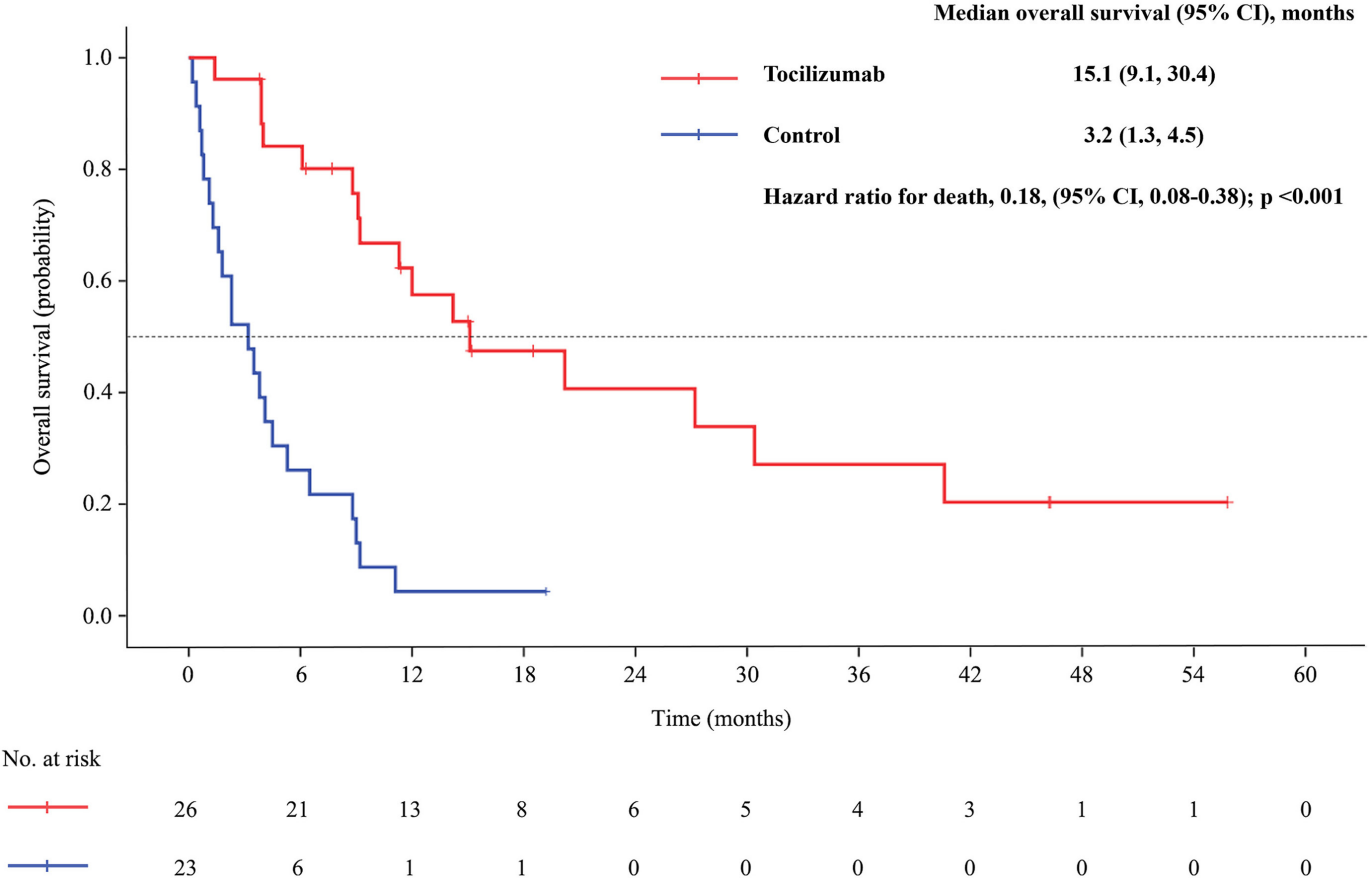
Kaplan–Meier curves for overall survival was compared between the tocilizumab group and control group.

As of the cut‐off date on 30 September 2023, the tocilizumab group recorded 16 patient deaths (61.5%), while the control group saw 22 deaths (95.7%). The median OS was markedly longer in the tocilizumab group, at 15.1 months (95% CI: 9.1–30.4 months), compared to 3.2 months in the control group (95% CI: 1.3–4.5 months; HR for death 0.18, 95% CI: 0.08–0.38; *p* < 0.001; Figure 2). Survival rates at 3 and 6 months stood at 96.2% and 52.2% in the tocilizumab group and 80.8% and 26.1% in the control group, respectively. A striking difference was observed in the number of patients with mGPS improvement at Week 12, with 23 out of 26 patients (88.5%) in the tocilizumab group showing improvement, compared to none in the control group (RD 0.88, 95% CI: 0.75–1.00; *p* < 0.001; Table 2).

**TABLE 2:** Efficacy endpoints.

	Combination of tocilizumab and antitumour therapy (*n* = 26)	Antitumour therapy alone (*n* = 23)	*p*
Primary endpoint^a^			
Overall survival (months)	15.1 (9.1–30.4)	3.2 (1.3–4.5)	< 0.001
Survival with mGPS improvement at Week 12	23 (88.5%)	0 (0)	< 0.001
Secondary endpoints^b^			
Body weight (kg)	5.15 ± 0.53	−5.69 ± 0.76	0.041
Albumin (g/dL)	5.89 ± 0.70	−2.97 ± 0.71	< 0.001
CRP (mg/L)	−91.50 ± 7.15	9.47 ± 13.69	< 0.001
mGPS	−1.61 ± 0.15	0.03 ± 0.08	< 0.001
Exploratory analysis endpoints^c^			
Appetite improvement	26 (100.0%)	6 (26.1%)	< 0.001
Fatigue improvement	26 (100.0%)	3 (13.0%)	< 0.001

Abbreviations: CRP, C‐reactive protein; mGPS, modified Glasgow Prognostic Score.

^a^ For primary endpoints, data are presented as median (95% CI) or *n* of patients with an event (%).

^b^ Secondary endpoints were changes from baseline over 12 weeks in body weight, mGPS, CRP and albumin. Least‐squares means, standard errors and *p* values were obtained from a mixed‐effects repeated measures model.

^c^ For exploratory analysis endpoints, data are presented as *n* of patients with an event (%).

**FIGURE 3:**
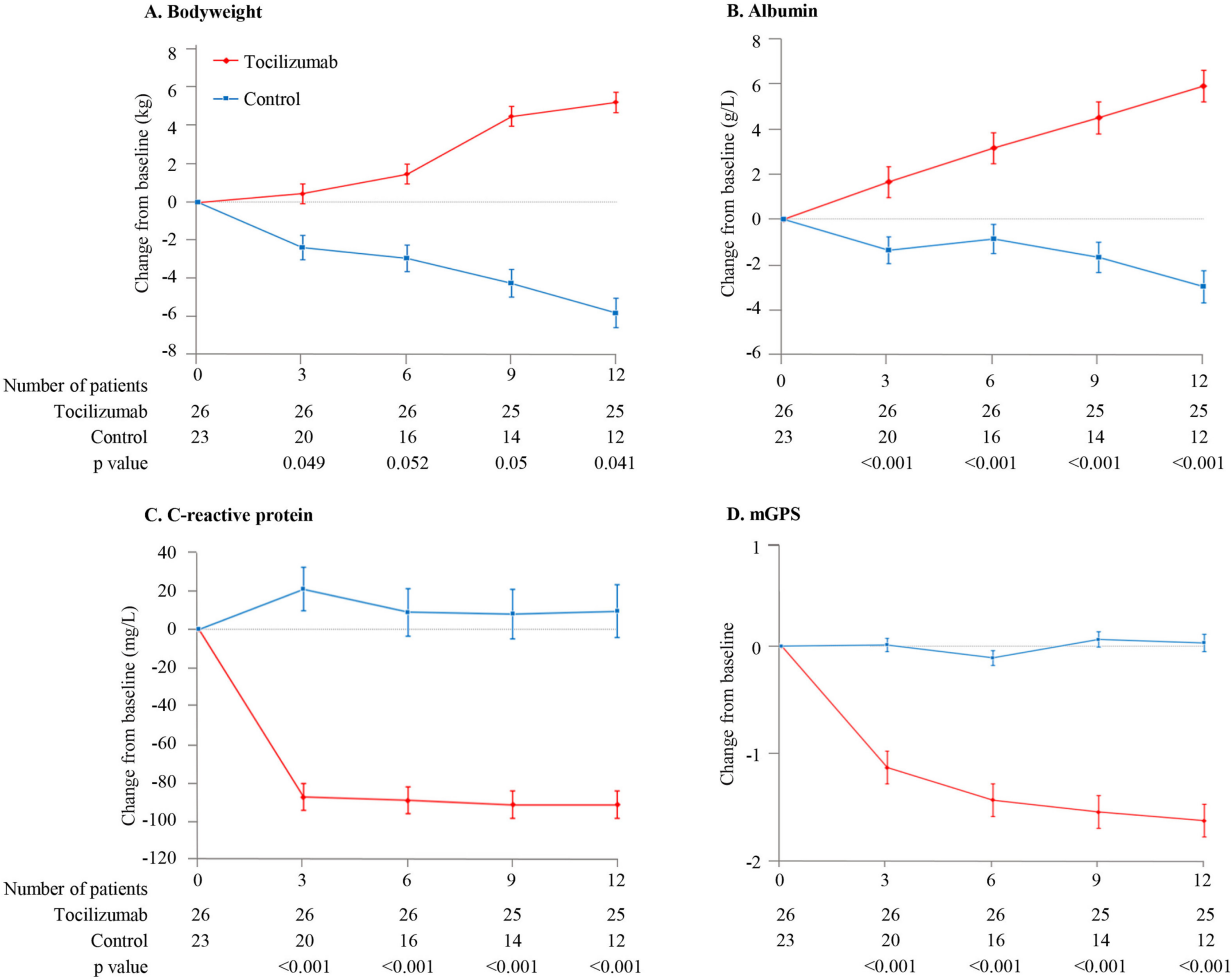
Change over time in secondary efficacy endpoints. The least‐squares mean (SE) change from baseline to each timepoint in (A) body weight, (B) albumin, (C) C‐reactive protein and (D) modified Glasgow Prognostic Score (mGPS).

Over the 12‐week study period, a significant increase in body weight was noted in the tocilizumab group (mean increase of 5.15 ± 0.53 kg) versus a decrease in the control group (mean decrease of −5.69 ± 0.76 kg; *p* = 0.041; Figure 3). Notably, significant improvements from baseline in body weight were observed in the tocilizumab group at Weeks 3 and 12, with no marked differences at Weeks 6 and 9. Additionally, patients receiving tocilizumab plus antitumour therapy showed significant improvements from baseline in albumin, C‐reactive protein and mGPS over 12 weeks, with notable differences emerging as early as Week 3 and persisting throughout the study period (Figure 3). The tocilizumab group also reported considerably higher rates of improvement in self‐reported appetite and fatigue compared to the control group (100.0% vs. 26.1%, *p* < 0.001 for appetite; 100% vs. 13.0%, *p* < 0.001 for fatigue; Table 2).

### 3.3 Prognostic Factors

Subgroup analyses revealed that the OS benefit associated with tocilizumab was particularly pronounced in all subgroups, except those with a baseline mGPS of 1 and histological types other than squamous or adenocarcinoma (Figure S2). Additionally, tocilizumab showed a superior rate of patients surviving with mGPS improvement at Week 12 in all subgroups (Figure S3). Stepwise Cox regression analysis identified tocilizumab as an independent protective factor for OS (HR for death 0.18, 95% CI: 0.09–0.38; *p* < 0.001; Table S2). Baseline ECOG PS of 2 and concurrent PD‐(L)1 inhibitor therapy were also recognized as independent protective factors for OS (HR 0.46, 95% CI: 0.23–0.92; *p* = 0.03; HR 0.47, 95% CI: 0.25–0.88; *p* = 0.03; Table S2). Further, stepwise logistic regression analysis showed tocilizumab as the sole independent protective factor for survival with mGPS improvement at Week 12 (*p* < 0.001; Table S2).

### 3.4 Safety

The overall incidence of adverse events was documented as 92.3% in patients receiving tocilizumab plus antitumour therapy and 100.0% in those receiving antitumour therapy alone. The incidence of Grade 3 or higher AEs was 34.6% with tocilizumab plus antitumour therapy, compared to 78.3% with antitumour therapy alone. No treatment‐related deaths were reported. The most common tocilizumab‐related adverse event was neutropenia (7.7%), followed by skin and subcutaneous tissue infections (3.8%). Tocilizumab discontinuation due to adverse events occurred in one patient (3.8%), and dose adjustments due to adverse events were necessary in two patients (7.7%). Given the variety of concurrent antitumour therapy regimens administered, confounding the interpretation of low‐frequency events, no statistical comparisons were made for the frequency of adverse events. However, patients receiving tocilizumab showed enhanced tolerability to antitumour treatments with a diminished occurrence of gastrointestinal adverse events. Although there was an observable trend towards a heightened incidence of neutropenia and thrombocytopenia (23.1% vs. 17.4% for neutropenia; 15.4% vs. 4.3% for thrombocytopenia), the frequencies of anaemia, lymphopenia and leukocytopenia were comparatively lower in the tocilizumab group (57.7% vs. 100% for anaemia; 15.4% vs 35.1% for lymphopenia; 23.1% vs. 30.4% for leukocytopenia; Table 3).

**TABLE 3:** Adverse events.

	Combination of tocilizumab and antitumour therapy (*n* = 26)	Antitumour therapy alone (*n* = 23)
Summary		
Any adverse events (AEs) (all grades)	24 (92.3%)	23 (100.0%)
Tocilizumab‐related adverse events (all grades)	3 (11.5%)	0 (0)
Grade 3 or worse adverse events	9 (34.6%)	18 (78.3%)
Tocilizumab‐related Grade 3 or worse events	3 (11.5%)	0 (0)
Any serious adverse events (SAE)	5 (19.2%)	20 (87.0%)
Tocilizumab‐related SAE	3 (11.5%)	0 (0)
AEs leading to death	0 (0)	0 (0)
Tocilizumab‐related AEs leading to death	0 (0)	0 (0)
AEs leading to discontinuation^a^	1 (3.8%)	0 (0)
AEs leading to dose adjustment^b^	2 (7.7%)	0 (0)
Select (by system organ class)^c^		
Gastrointestinal system		
Vomiting	1 (3.8%)	6 (26.1%)
Abdominal pain	3 (11.5%)	5 (21.7%)
Diarrhoea	1 (3.8%)	5 (21.7%)
Constipation	9 (34.6%)	13 (56.5%)
Liver enzyme increase	9 (34.6%)	16 (69.6%)
Lipase increase	1 (3.8%)	0 (0)
Respiratory system		
Haemoptysis	1 (3.8%)	3 (13.0%)
Dyspnoea	6 (23.1%)	15 (65.5%)
Skin		
Rash	2 (7.7%)	3 (13.0%)
Blood and lymphatic system		
Anaemia	15 (57.7%)	23 (100.0%)
Leukocytosis	0 (0)	1 (4.3%)
Leukocytopenia	6 (23.1%)	7 (30.4%)
Neutropenia	6 (23.1%)	4 (17.4%)
Lymphopenia	4 (15.4%)	9 (39.1%)
Thrombocytopenia	4 (15.4%)	1 (4.3%)
Endocrine		
Hypothyroidism	1 (3.8%)	2 (8.7%)
Renal		
Acute kidney injury	2 (7.7%)	1 (4.3%)
Infection		
Sepsis	0 (0)	2 (8.7%)
Pneumonia	2 (7.7%)	1 (4.3%)
Urinary tract infection	1 (3.8%)	1 (4.3%)
Skin and subcutaneous tissue infection	1 (3.8%)	0 (0)
Metabolism and nutrition		
Hyponatremia	4 (15.4%)	14 (60.9%)
Hypochloridaemia	0 (0)	3 (13.0%)
Hypocalcaemia	1 (3.8%)	4 (17.4%)
Hypercalcaemia	1 (3.8%)	2 (8.7%)
Hypokalaemia	3 (11.5%)	9 (39.1%)
Hyperglycaemia	0 (0)	4 (17.4%)

*Note:* Data presented as *n* of patients with an event (%).

^a^ One tocilizumab‐related adverse event resulted in drug discontinuation due to a skin and subcutaneous tissue infection.

^b^ Two dose reductions were reported in the tocilizumab group due to neutropenia.

^c^ Selected adverse events are those with a potential treatment‐related aetiology that require frequent monitoring/intervention.

## 4 Discussion

To the best of our knowledge, this study represents the inaugural clinical investigation into the efficacy and safety of tocilizumab for advanced NSCLC with concomitant cachexia and elevated plasma IL‐6 levels. The combination of tocilizumab and antitumour therapy demonstrated a significant clinical advantage, yielding a statistically notable improvement in OS compared to antitumour therapy alone. In the tocilizumab group, a significant increase in body weight (mean increase of 9.8%) was observed by Week 12, surpassing the weight gain observed with ghrelin therapy (1.5%–3.2%), which has been approved for clinical use in Japan [18, 19]. This group also exhibited rapid and sustained improvements in inflammatory and nutritional markers, including albumin, CRP and mGPS, starting as early as Week 3 and persisting throughout the 12‐week period. An exploratory analysis indicated a reduction in self‐reported symptom burden over the same timeframe in the tocilizumab group.

Numerous clinical studies have, thus far, failed to demonstrate significant survival benefits in the context of cancer cachexia [18, 19, 20, 21, 22, 23]. This shortfall may be attributed to inadequacies in comprehending the pathophysiological mechanisms governing cachexia, potentially leading to suboptimal study designs and inappropriate patient selection in prior clinical investigations. Such limitations have likely influenced the observed survival outcomes negatively. The heterogeneity of cachexia's underlying mechanisms suggests that a one‐size‐fits‐all approach may be overly simplistic. IL‐6, a proinflammatory cytokine strongly linked to weight loss, is increasingly recognized as a critical factor in the development of cancer cachexia [5, 10, 24]. Our research distinctly targeted cachexia with elevated IL‐6 and CRP levels, thereby focusing on a specific subset of this multifaceted condition. Furthermore, our study deviated from the conventional approach in cachexia trials by prioritizing OS as the primary endpoint [25]. This shift is pivotal for a comprehensive evaluation of the efficacy of anticachexia agents. Additionally, we incorporated a composite endpoint assessing survival coupled with improvement in the mGPS at Week 12, thereby evaluating both survival and alterations in inflammation and nutritional status [17].

In contrast to prevailing findings in oncological research, which consistently identify higher ECOG PS and significant weight loss as negative prognostic and predictive indicators [26, 27], our investigation demonstrates a notable survival advantage in OS conferred by the addition of tocilizumab. This survival benefit is particularly pronounced in patients experiencing a weight loss of 5%–10% and presenting with an ECOG PS of 2. This phenomenon may be attributed to tocilizumab's role in mitigating cancer‐associated inflammation, which is more severe in patients exhibiting weight loss and higher ECOG PS scores. Additionally, tocilizumab appears to enhance tolerance to antitumour treatments, thereby offering a substantial survival benefit in this patient subset, which historically demonstrates limited treatment tolerance and suboptimal responses to conventional therapies [28, 29, 30, 31, 32]. Moreover, our study reveals that patients who have undergone more than one prior antitumour therapy regimen derive greater survival benefits from tocilizumab administration. This may be due to tocilizumab's potential in reversing resistance to chemotherapy or targeted therapies through the inhibition of IL‐6 signalling [13, 14, 33, 34]. In a similar vein, the synergistic interaction between IL‐6 blockade and PD‐(L)1 inhibition could elucidate the observed enhanced benefit in patients receiving PD‐(L)1 inhibitor therapy in conjunction with tocilizumab [15, 35, 36, 37, 38, 39].

In this study, the addition of tocilizumab notably appeared to diminish the adverse events associated with antitumour treatments. Compared to the control group, the tocilizumab cohort experienced an 8% reduction in all‐grade adverse events and a 40% decrease in Grades 3 and 4 adverse events. This diminution may be attributed to tocilizumab's role in resolving both cancer‐related and antitumour treatment‐induced inflammation. Intriguingly, tocilizumab significantly decreased the incidence of anaemia, while concomitantly increasing the rate of neutropenia. The amelioration of anaemia could be linked to the alleviation of inflammation, in which cytokines restrict erythropoiesis both directly and indirectly and shorten the erythrocyte lifespan [40, 41, 42]. Similarly, the rise in neutropenia rates might be a result of tocilizumab's effect on resolving myeloid inflammation, as indicated by the concurrent increased lymphocyte ratio, potentially synergizing with antitumour therapies to confer a survival advantage, distinct from myelosuppression typically induced by chemotherapy. This hypothesis is further supported by the observed improvement in leukocytopenia. Nonetheless, careful consideration is warranted when combining tocilizumab with chemotherapy due to the potential induction of Grade 3/4 neutropenia, as evidenced in two instances within our study. Both cases of severe neutropenia occurred after administering an 8‐mg/kg dose of tocilizumab, with no subsequent occurrences following a dose reduction to 6 mg/kg.

This study is subject to several limitations that merit careful consideration. Firstly, while the qualitative analysis suggested improvements in anorexia and fatigue among a majority of patients treated with tocilizumab, quantitative assessment of changes in cachexia‐related symptoms using structured inventories or handgrip strength measurements was not conducted. This omission limits the ability to objectively measure symptom alleviation. Secondly, this study focused only on changes in body weight, a parameter potentially affected by oedema, rather than on direct measurements of body composition such as lean body mass, skeletal muscle index, visceral adipose tissue and subcutaneous adipose tissue. Thirdly, the retrospective design of the study resulted in heterogeneity in the antitumour treatment regimens between the two groups. Although statistically controlled, this heterogeneity could introduce potential biases. Moreover, the limited sample size did not perfectly meet the events‐per‐variable requirement for stepwise multivariable regression, potentially undermining the robustness of the findings. Finally, the single‐centre nature of this study significantly restricts the generalizability of our results. These aspects highlight the importance of conducting future multicentre, large‐scale studies to confirm and extend these preliminary results. Based on our retrospective findings, we have initiated an investigator‐led Phase II clinical trial (ChiCTR2400080103) to evaluate the safety and efficacy of tocilizumab in patients with solid tumours, concomitant cachexia and elevated plasma IL‐6 levels.

In conclusion, for patients with NSCLC accompanied by cachexia and elevated plasma IL‐6 levels, the addition of tocilizumab to standard anticancer treatments appears to mitigate the effects of cancer cachexia and extend survival, while also enhancing tolerance to conventional oncologic therapies. Overall, tocilizumab emerges as a safe and efficacious therapeutic option for managing advanced NSCLC with concomitant IL‐6‐elevated cachexia.

## Ethics Statement

The study was conducted in strict accordance with the ethical principles of the Declaration of Helsinki. The off‐label use of tocilizumab was approved by the joint session of the medical committee, pharmacy management and therapeutics committee and institutional review board of Peking Union Medical College Hospital. Informed consent was obtained from all the patients receiving tocilizumab therapy. Patient data and treatment information were collected with the approval of the institutional review board (No. I‐23PJ769).

## Conflicts of Interest

The authors declare no conflicts of interest.

## Supporting information


**Figure S1.** Clinical practice for the diagnosis of IL‐6‐elevated cachexia.
**Figure S2.** Forest plots showing hazard ratios of tocilizumab relative to control for overall survival in different subgroups.
**Figure S3.** Forest plots showing odds ratios of tocilizumab relative to control for survival with mGPS improvement at Week 12 in different subgroups.
**Table S1.** Summary of concurrent antitumour regimens.
**Table S2.** Stepwise multivariate regression analyses for primary outcomes.

## Data Availability

The datasets used and analysed during the current study are available from the corresponding authors on reasonable request.
