# Supplementary material for: Tocilizumab for Advanced Non‐Small‐Cell Lung Cancer With Concomitant Cachexia: An Observational Study
Source: J Cachexia Sarcopenia Muscle. 2024 Nov 11;15(6):2815–25. doi: 10.1002/jcsm.13638 (PMC11634525; doi:10.1002/jcsm.13638)
Supplement: Supplementary file 1 — Figure S1. Clinical practice for the diagnosis of IL‐6‐elevated cachexia. Figure S2. Forest plots showing hazard ratios of tocilizumab relative to control for overall survival in different subgroups. Figure S3. Forest plots showing odds ratios of tocilizumab relative to control for survival with mGPS improvement at Week 12 in different subgroups. Table S1. Summary of concurrent antitumour regimens. Table S2. Stepwise multivariate regression analyses for primary outcomes. [file JCSM-15-2815-s001.docx]

**Supplementary Materials**

Tocilizumab for Advanced Non-Small Cell Lung Cancer with Concomitant Cachexia: An Observational Study

**Figure S1.** Clinical practice for the diagnosis of IL-6-elevated cachexia

**Figure S2.** Forest plots showing hazard ratios of tocilizumab relative to control for overall survival in different subgroups

**Figure S3.** Forest plots showing odds ratios of tocilizumab relative to control for survival with mGPS improvement at week 12 in different subgroups

**Table S1.** Summary of concurrent anti-tumour regimens

**Table S2.** Stepwise multivariate regression analyses for primary outcomes

**Figure S1. Clinical practice for the diagnosis of IL-6-elevated cachexia**


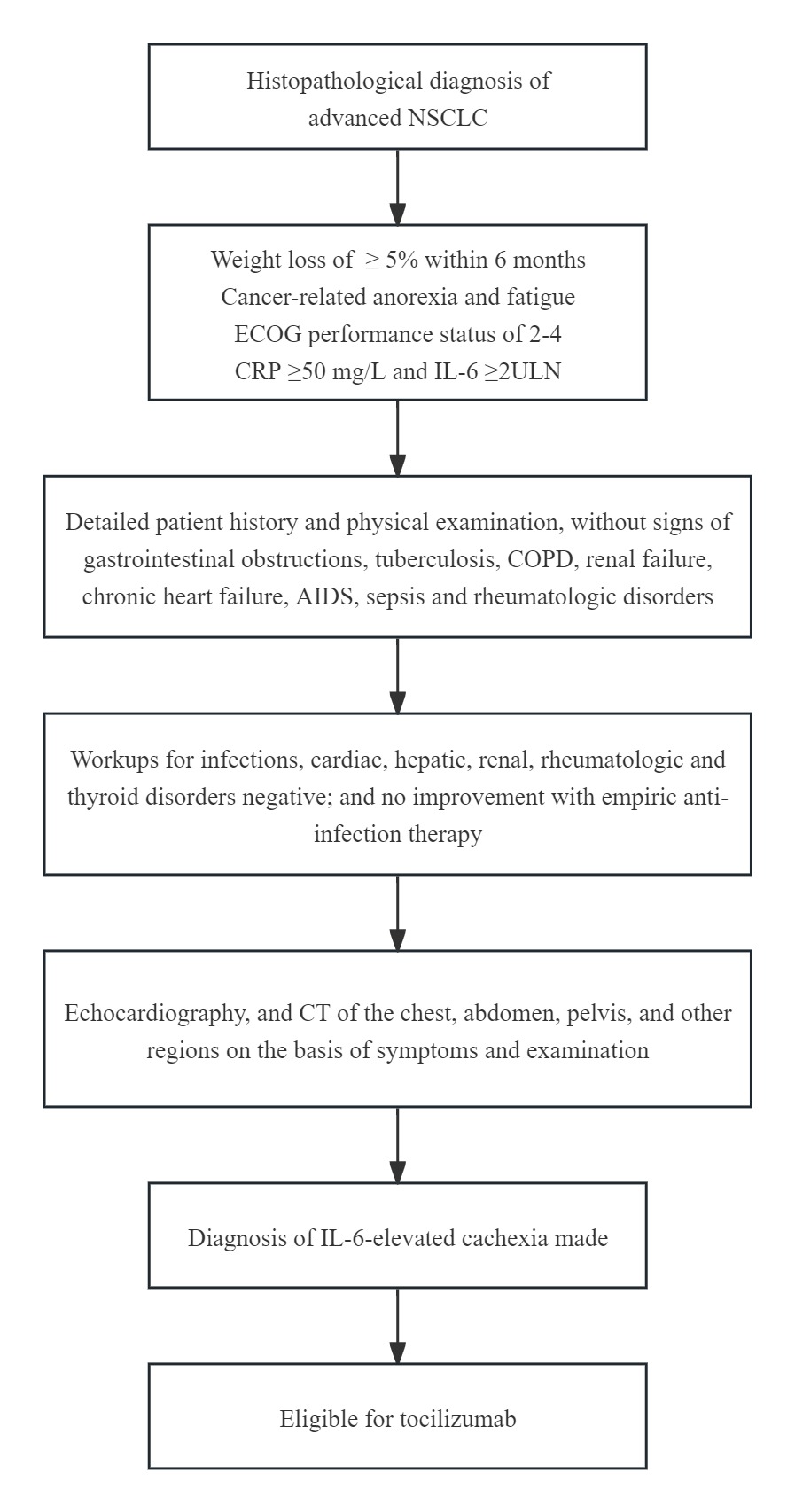


Abbreviations: AIDS, acquired immunodeficiency syndrome; CT, computed tomography; COPD, chronic obstructive pulmonary disease; ECOG, Eastern Cooperative Oncology Group; NSCLC, non-small cell lung cancer; ULN, upper limit of normal.

**Figure S2. Forest plots showing hazard ratios of tocilizumab relative to control for death in different subgroups**


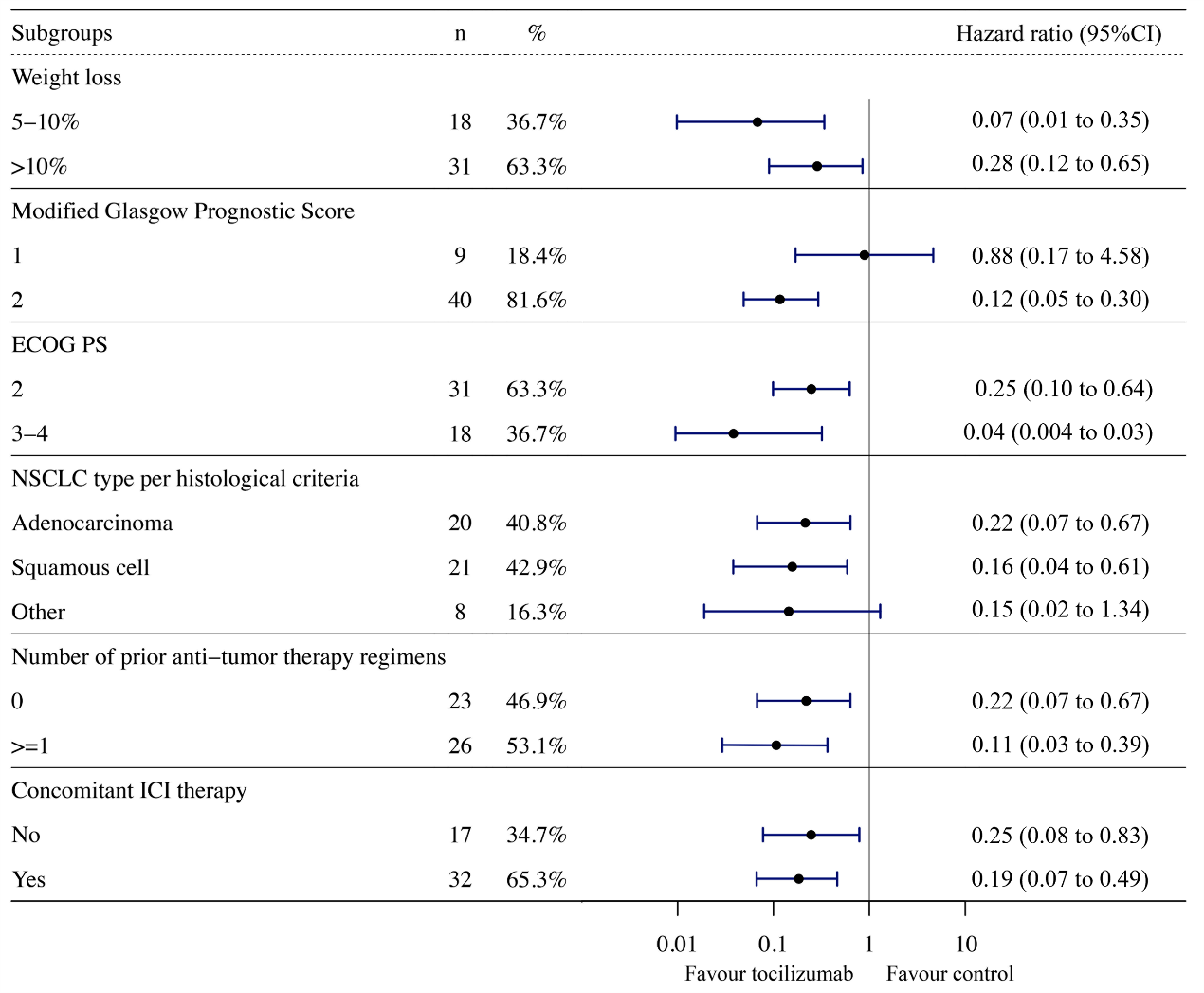


Abbreviations: CI, confidence interval; ECOG PS, Eastern Cooperative Oncology Group performance status; NSCLC, non-small cell lung cancer; PD-(L)1, programmed cell death protein (ligand) 1.

**Figure S3. Forest plots showing risk differences of tocilizumab relative to control for survival with mGPS improvement at week 12 in different subgroups**


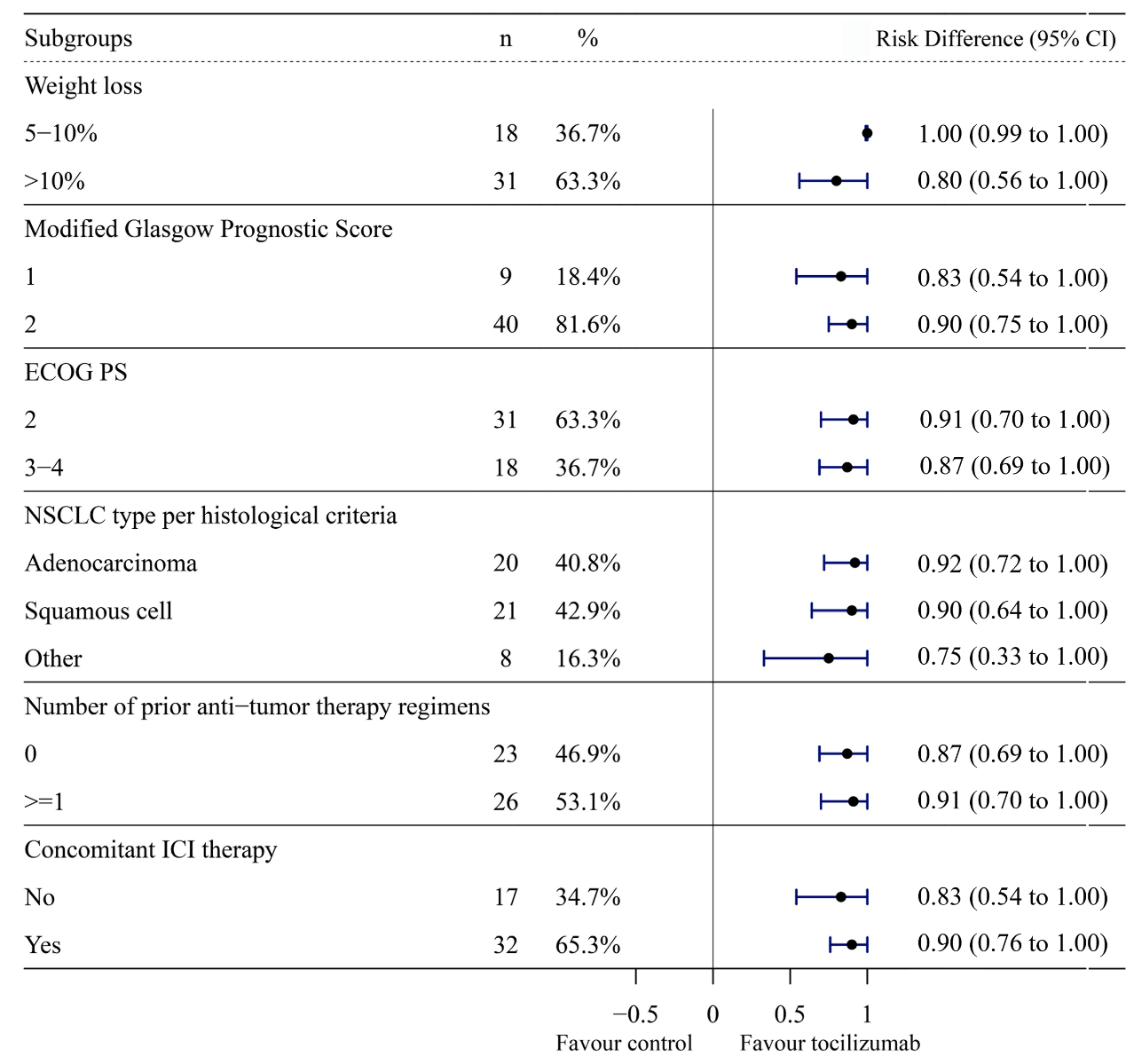


Abbreviations: CI, confidence interval; ECOG PS, Eastern Cooperative Oncology Group performance status; NSCLC, non-small cell lung cancer; PD-(L)1, programmed cell death protein (ligand) 1.

**Table S1. Summary of concurrent anti-tumour regimens**

|  | Combination of tocilizumab and anti-tumour therapy (n=26) | Anti-tumour therapy alone (n=23) |
| --- | --- | --- |
| Monotherapy |  |  |
| Pembrolizumab | 1 (3.8%) | 1 (4.3%) |
| Osimertinib | 0 (0) | 1 (4.3%) |
| Furmonertinib | 1 (3.8%) | 0 (0) |
| Anlotinib | 1 (3.8%) | 1 (4.3%) |
| Gemcitabine | 0 (0) | 2 (8.7%) |
| Albumin-Bound Paclitaxel and Carboplatin | 0 (0) | 1 (4.3%) |
| Paclitaxel and Carboplatin | 0 (0) | 1 (4.3%) |
| Vinorelbine and Ifosfamide | 0 (0) | 1 (4.3%) |
| Combination therapy |  |  |
| Gemcitabine and Pembrolizumab | 0 (0) | 1 (4.3%) |
| Docetaxel and Pembrolizumab | 0 (0) | 1 (4.3%) |
| Albumin-Bound Paclitaxel and Pembrolizumab | 4 (15.4%) | 0 (0) |
| Paclitaxel Liposome and Pembrolizumab | 2 (7.7%) | 0 (0) |
| Pemetrexed and Envafolimab | 1 (3.8%) | 0 (0) |
| Pemetrexed, Carboplatin and Pembrolizumab | 2 (7.7%) | 2 (8.7%) |
| Pemetrexed, Carboplatin and Tislelizumab | 1 (3.8%) | 0 (0) |
| Paclitaxel, Carboplatin and Pembrolizumab | 0 (0) | 3 (13.0%) |
| Albumin-Bound Paclitaxel, Carboplatin and Pembrolizumab | 4 (15.4%) | 3 (13.0%) |
| Albumin-Bound Paclitaxel , Carboplatin and Tislelizumab | 2 (7.7%) | 0 (0) |
| Pemetrexed, Carboplatin and Bevacizumab | 2 (7.7%) | 0 (0) |
| Pemetrexed, Cisplatin and Bevacizumab | 0 (0) | 2 (8.7%) |
| Docetaxel, Nedaplatin and Erlotinib | 0 (0) | 1 (4.3%) |
| Pemetrexed, Carboplatin and Anlotinib | 1 (3.8%) | 0 (0) |
| Docetaxel and Anlotinib | 1 (3.8%) | 0 (0) |
| Pembrolizumab and Anlotinib | 1 (3.8%) | 0 (0) |
| Albumin-Bound Paclitaxel, Pembrolizumab and Anlotinib | 1 (3.8%) | 1 (4.3%) |
| Albumin-Bound Paclitaxel, Carboplatin, Tislelizumab, and Bevacizumab | 1 (3.8%) | 0 (0) |
| Other | 0 (0) | 1 (4.3%) |

Concurrent anti-tumour therapy in the tocilizumab group included any systemic therapy used on or after the date of the first dose of tocilizumab and up to and including 7 days after the date of the last dose. In the control group, it included any systemic therapy used after the diagnosis of inflammatory cachexia.

**Table S2. Stepwise** **multivariate regression analyses for primary outcomes**

|  | Reference | **HR (95% CI)** | P value |
| --- | --- | --- | --- |
| **Overall survival** |  |  |  |
| Tocilizumab | No tocilizumab | 0.18 (0.09 to 0.38) | <0.001 |
| ECOG PS of 2 | ECOG PS of 3-4 | 0.46 (0.23 to 0.92) | 0.03 |
| Concomitant PD-(L)1 inhibitor therapy | No PD-(L)1 inhibitor therapy | 0.47 (0.25 to 0.88) | 0.03 |
|  | Reference | **OR (95% CI)** | P value |
| **Survival with mGPS improvement at week 12** | |  |  |
| Tocilizumab | No tocilizumab | Not estimated | <0.001 |

Abbreviations: CI, confidence interval; ECOG PS, Eastern Cooperative Oncology Group performance status; HR, hazard ratio; mGPS, modified Glasgow Prognostic Score; PD-(L)1, programmed cell death protein (ligand) 1; OR, odd ratio.
